# Cardiac magnetic resonance dual bolus myocardial perfusion quantification superior to the single bolus analysis method

**DOI:** 10.1186/1532-429X-17-S1-P58

**Published:** 2015-02-03

**Authors:** Kaatje Goetschalckx, Piet Claus, Jan Bogaert, Attila Tóth, Béla Merkely, Stefan Janssens, Frank E Rademakers

**Affiliations:** 1 Division of Cardiovascular Diseases, University Hospitals Leuven, Leuven, Belgium; 2 Department of Cardiovascular Sciences, University Hospitals Leuven, KU Leuven, Leuven, Belgium; 3 Department of Radiology, University Hospitals Leuven, Leuven, Belgium; 4 Heart and Vascular Center, Semmelweis University, Budapest, Hungary

## Background

Quantitative assessment of myocardial blood flow (MBF) with first-pass perfusion cardiac magnetic resonance (CMR) is subject to disparity in methodology. The aim of this study was to compare single (SB) and dual bolus (DB) analysis methods in a large patient population with recently revascularized myocardial infarction.

## Methods

In this substudy of the NOMI-trial (ClinicalTrials.gov identifier: NCT01398384), CMR rest and adenosine stress perfusion were analyzed in 119 patients at 4 months after acute myocardial infarction with TIMI 2-3 flow after primary PCI. A balanced turbo gradient echo sequence with non-shared prepulse was used in 1.5 tesla MR scanner (Achieva, Philips Medical Systems). MBF was quantified using Fermi deconvolution with SB (0.05 mmol/kg) and DB (equal volumes of 0.0027 mmol/kg followed by 0.05 mmol/kg of contrast agent) in 6 segments of basal and midventricular short axis perfusion slices. Because of the increased movement and time consuming nature of the analysis, apical slices were excluded. Segments were grouped according to the presence of no (late gadolinium enhancement LGE=0), little (LGE<50%) or more then 50% of infarct scar (LGE≥50%) on corresponding LGE-images. Myocardial perfusion reserve (MPR) was calculated by dividing stress by rest MBF. In a subset of 5 patients, perfusion analysis of the midventricular slice at rest and stress was repeated by the same (KG) and another observer (PC), both blinded to the first results, to obtain intra- and interobserver variability respectively, by calculating the coefficients of variation (COV).

## Results

**Figure 1:**
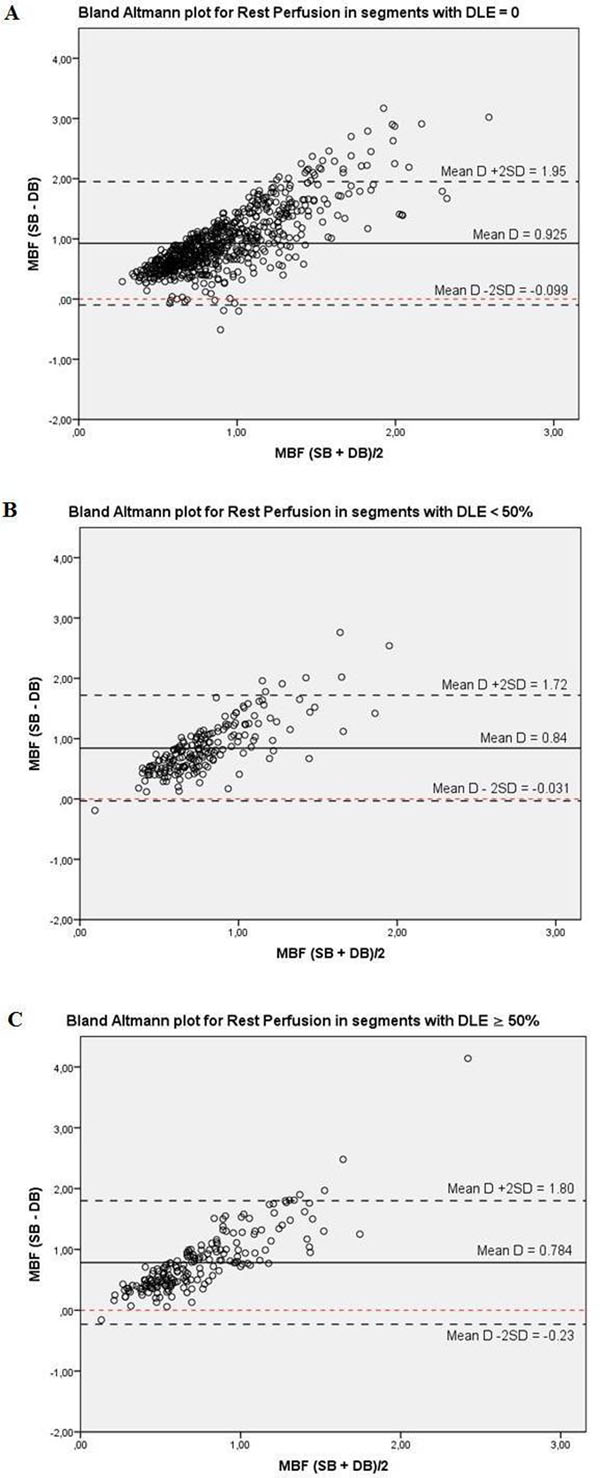


Two hundred thirthy eight slices with a total of 1428 segments were analyzed for rest and stress perfusion with both methods. MBF quantification is feasible in infarcted and non-infarcted segments with SB and DB, with at least 84% of segments being analyzable and a good correlation (r = 0.81, p<0.01) between methods. MBF decreases significantly in segments with increasing infarct size, both at rest and during stress. MPR also decreases significantly with increasing infarct size. (cf. Table 1) MBF values with SB are high due to saturation effects even at a dose of 0.05 mmol/kg. (cf. Figure 1)

**Table 1:** 

MBF**(ml/g/min)**	LGE = 0	LGE < 50	LGE ≥ 50	p	p	p
mean ± SD (n)	1	2	3	1-2	2-3	1-3

REST						

SB	1.36 ± 0.58 (741)	1.22 ± 0.47 (199)	1.12 ± 0.58 (202)	< 0.05	< 0.05	< 0.05

DB	0.43 ± 0.21 (719)	0.37 ± 0.18 (191)	0.35 ± 0.18 (196)	< 0.05	0.09	< 0.05

STRESS						

SB	3.22 ± 1.22 (683)	2.68 ± 1.16 (190)	2.21 ± 1.14 (205)	< 0.05	< 0.05	< 0.05

DB	1.33 ± 0.57 (688)	1.08 ± 0.54 (192)	0.88 ± 0.47 (204)	< 0.05	< 0.05	< 0.05

MPR						

SB	2.69 ± 1.45 (624)	2.39 ± 1.29 (176)	2.30 ± 1.57 (177)	< 0.05	ns	< 0.05

DB	3.65 ± 1.93 (609)	3.36 ± 1.99 (173)	2.98 ± 1.67 (175)	< 0.05	ns	< 0.05

MPR = myocardial perfusion reserve = MBF stress/MBF rest

Inter- and intra-observer variability of DB is smaller than for SB, with an inter-observer COV of respectively 16 and 24% for rest perfusion, and 14 and 21% for stress perfusion, and an intra-observer COV of respectively 16 and 19% for rest perfusion, and 20 and 21% for stress perfusion.

## Conclusions

Quantification of rest and stress perfusion with DB is superior to SB, because the SB method is more subject to saturation effects and analysis reproducibility is better for DB.

## Funding

No disclosures.

